# The role of serotonin 5-HT_2A_ receptors in memory and cognition

**DOI:** 10.3389/fphar.2015.00225

**Published:** 2015-10-06

**Authors:** Gongliang Zhang, Robert W. Stackman

**Affiliations:** ^1^College of Basic Medicine, Anhui Medical UniversityHefei, China; ^2^Department of Biology, Charles E. Schmidt College of Science, Florida Atlantic University, JupiterFL, USA; ^3^Jupiter Life Science Initiative, Florida Atlantic University, JupiterFL, USA; ^4^Department of Psychology, Charles E. Schmidt College of Science, Florida Atlantic University, JupiterFL, USA

**Keywords:** serotonin, 5-HT_2A_ receptor, learning, memory, cognition

## Abstract

Serotonin 5-HT_2A_ receptors (5-HT_2A_Rs) are widely distributed in the central nervous system, especially in brain region essential for learning and cognition. In addition to endogenous 5-HT, several hallucinogens, antipsychotics, and antidepressants function by targeting 5-HT_2A_Rs. Preclinical studies show that 5-HT_2A_R antagonists have antipsychotic and antidepressant properties, whereas agonist ligands possess cognition-enhancing and hallucinogenic properties. Abnormal 5-HT_2A_R activity is associated with a number of psychiatric disorders and conditions, including depression, schizophrenia, and drug addiction. In addition to its traditional activity as a G protein-coupled receptor (GPCR), recent studies have defined novel operations of 5-HT_2A_Rs. Here we review progress in the (1) receptor anatomy and biology: distribution, signaling, polymerization and allosteric modulation; and (2) receptor functions: learning and memory, hallucination and spatial cognition, and mental disorders. Based on the recent progress in basic research on the 5-HT_2A_R, it appears that post-training 5-HT_2A_R activation enhances non-spatial memory consolidation, while pre-training 5-HT_2A_R activation facilitates fear extinction. Further, the potential influence that 5-HT_2A_R-elicited visual hallucinations may have on visual cue (i.e., landmark) guided spatial cognition is discussed. We conclude that the development of selective 5-HT_2A_R modulators to target distinct signaling pathways and neural circuits represents a new possibility for treating emotional, neuropsychiatric, and neurodegenerative disorders.

## Introduction

The serotonin (5-HT) 5-HT_2A_ receptor (5-HT_2A_R) is a GPCR of the type A family. It was defined as the classical D receptor initially by Gaddum and Picarelli (1957), and later referred as the 5-HT_2_ receptor by Peroutka and Snyder (1979). The 5-HT_2A_R gene is located on human chromosome 13q14-q21. *HTR2A* gene codes for a 471-amino acid sequence in rat, mouse, and human (Sparkes et al., 1991). The rat 5-HT_2A_R was cloned in 1988 (Pritchett et al., 1988) and the human 5-HT_2A_R was reported by Julius et al. (1990). Central 5-HT_2A_Rs exert diverse behavioral, physiological, and psychological influences (Hoyer et al., 2002; Hannon and Hoyer, 2008; Homberg, 2012). Abnormality in the structure and function of the 5-HT_2A_R is associated with a number of disorders, including schizophrenia, depression/anxiety, and drug addiction. Furthermore, many hallucinogenic drugs exert their psychoactive effects by acting as agonists for 5-HT_2A_Rs. Preclinical studies show that 5-HT_2A_R blockade has antipsychotic (Meltzer, 1999), antidepressant (Kroeze and Roth, 1998; Roth et al., 1998) and anxiolytic properties (Cohen, 2005). Pharmacological studies indicate that high-affinity antagonists of 5-HT_2A_Rs are effective atypical antipsychotics, due to their demonstrated efficacy to reduce both positive and negative symptoms of schizophrenia. Results from recent molecular biological and neuropharmacological studies suggest some exciting potential new avenues by which 5-HT_2A_Rs influence CNS function. Here we review progress in understanding the contribution of 5-HT_2A_Rs to modulation of learning and memory through an analysis of their (1) anatomy and biology: distribution, signaling, polymerization, and allosteric modulation; and (2) functions: learning and memory, hallucination and spatial cognition, and mental disorders. Based on the recent progress in 5-HT_2A_R research, we suggest that selective 5-HT_2A_R modulators targeting distinct signaling pathways may hold significant efficacy as new therapeutic approaches for several neurological disorders that present with cognitive impairment.

## 5-HT_2A_R Anatomy and Biology in CNS

### Cellular and Subcellular Distribution

Serotonin 5-HT_2A_Rs are widely distributed in the CNS. In the rat brain, immunohistochemical studies show that 5-HT_2A_Rs are broadly expressed in the cerebral cortex – especially in layers I and IV–V, the piriform and entorhinal cortex, the claustrum, endopiriform nucleus, and olfactory bulb/anterior olfactory nucleus, brainstem, as well as the limbic system and the basal ganglia; especially in the NAc and caudate nucleus (Xu and Pandey, 2000; Hannon and Hoyer, 2008). Interestingly, 5-HT_2A_R binding appears to be absent from cerebellum (Xu and Pandey, 2000).

In human brain, autoradiographic analysis using [^3^H] ketanserin indicates a high density of 5-HT_2A_R binding in laminae III and V of the frontal, parietal, temporal, occipital, anterogenual cortexes, and entorhinal area. 5-HT_2A_Rs are also visualized in the mammillary bodies of the hypothalamus, claustrum, and the lateral nucleus of the amygdala. The hippocampus, caudate, putamen, and accumbens nuclei present an intermediate density of binding. Areas such as the thalamus, brain stem, cerebellum and spinal cord contained only low to very low densities of binding (Pazos et al., 1987). *In situ* hybridization studies reveal that 5-HT_2A_R mRNA is present in all neocortical areas, especially in layer 5 pyramidal neurons, and in putative interneurons. 5-HT_2A_R mRNA was observed at minimal levels in the hippocampus and not in the raphe, cerebellum, substantia nigra or striatum (Burnet et al., 1995).

Morphological and double immunofluorescence analyses confirmed the presence of 5-HT_2A_Rs on pyramidal neurons, interneurons, and glial cells in neocortex, amygdala and hippocampus (Willins et al., 1997; Bombardi, 2012, 2014). Thus, predicting the functional influence of activated cortical 5-HT_2A_Rs is not straightforward, since these receptors would be capable of direct excitation and modulating feed-forward inhibition. In addition, 5-HT_2A_Rs are located on cholinergic (Quirion et al., 1985) and glutamatergic neurons (Hasuo et al., 2002). 5-HT_2A_R immunolabeling was also observed on glial cells in many forebrain regions: astrocytes were identified by double immunolabeling as cells in which 5-HT_2A_R and GFAP was colocalized (Xu and Pandey, 2000); and on microglia (Glebov et al., 2015). These findings demonstrate that consideration of the serotonin-mediated signaling at 5-HT_2A_Rs must include pathways that involve neurons and glial cells alike. It will be of interest to determine the degree to which functional influences expressed by the activation of 5-HT_2A_Rs are dependent upon neurons, astrocytes, and microglial cells, and to determine whether clinically relevant features of 5-HT_2A_Rs are related to changes in neurons or astrocytes.

At the subcellular level, 5-HT_2A_R immunolabeling is found on cell bodies and processes of neurons (Cornea-Hebert et al., 1999; Xu and Pandey, 2000); in particular, at both pre- and post-synaptic compartments (Miner et al., 2003). However, the majority of evidence suggests a predominant expression at postsynaptic dendritic spines and shafts of non-5-HT neurons. Our own immuno-electron microscopy data revealed that 5-HT_2A_R is also distributed in the dendritic spines, shafts, and presynaptic terminals of CA1 neurons in the mouse dorsal hippocampus (Zhang et al., 2015). Consideration should also be given to evidence suggesting that 5-HT_2A_R subunits are extensively and dynamically trafficked between the cytoplasm and the neuronal membrane, as much 5-HT_2A_R label has been identified at cytoplasmic rather than membrane bound compartments in adult rat neocortex (Cornea-Hebert et al., 1999). It will be of interest to determine the corresponding function of 5-HT_2A_R subunit trafficking between the respective neuronal sub-compartments, and the intracellular signaling that promotes trafficking.

### Interacting Proteins

Multiple interacting proteins regulate the function of 5-HT_2A_Rs in the membrane. 5-HT_2A_Rs interact with multiple PDZ protein-1 (MUPP1) and PSD-95 PDZ proteins (Jones et al., 2009). The 5-HT_2A_R colocalizes with PSD-95 and with MUPP1 in a subset of dendritic spines of rat cortical pyramidal neurons. PDZ proteins are vital for docking 5-HT_2A_R to the dendrites in cortical neurons and preventing the internalization of 5-HT_2A_Rs (Xia et al., 2003). MUPP1 is enriched in dendritic spine PSD domains of pyramidal neurons and enhances the localization of 5-HT_2A_R to the cell surface. Within cortical pyramidal neurons, PSD-95 regulates the functional activity of 5-HT_2A_R by promoting apical dendritic targeting and stabilizing receptor turnover. The complex of 5-HT_2A_R and PSD-95 plays a key role in 5-HT_2A_R-mediated head-twitch behavior in mice (Abbas et al., 2009). Binding of calmodulin to the 5-HT_2A_R C-terminus impedes PKC-mediated phosphorylation of the 5-HT_2A_R, thus, preventing its desensitization (Turner and Raymond, 2005). Conversely, association of p90-RSK2 with 5-HT_2A_R (intracellular 3 loop) silences the GPCR’s signaling (Sheﬄer et al., 2006). Caveolin-1 interacts with 5-HT_2A_R and profoundly modulates its signaling by facilitating the interaction of 5-HT_2A_R with Gαq (Bhatnagar et al., 2004). 5-HT_2A_R and the light chain 2 domain of the microtubule-associated protein MAP1A are co-localized in the intracellular compartment of pyramidal neuronal dendrites of adult rats and may participate in intraneuronal signaling processes involving cytoskeletal elements (Cornea-Hebert et al., 2002). In consideration of these properties, we suggest that altering 5-HT_2A_R-coupled proteins and pathways may enable an alternative method to selectively promote distinct modulatory functions of 5-HT_2A_Rs.

### Signaling

Activation of neuronal 5-HT_2A_Rs can induce pleiotropic effects via G protein-dependent, ligand-dependent, and ligand-independent signaling pathways, including phospholipase signaling, ERK pathway, and tyrosine kinase pathway in neurons (Millan et al., 2008; Masson et al., 2012). In most circumstances, activation of 5-HT_2A_Rs increases intracellular Ca^2+^ levels via G_αq_-PLC-IP3 signaling (Hagberg et al., 1998). In PFC, activation of 5-HT_2A_Rs suppresses membrane Ca_v_1.2 L-type Ca^2+^ currents via a G_αq_-mediated PLCβ/IP_3_/calcineurin signaling pathway (Day et al., 2002). 5-HT_2A_R activation also stimulates the G_α12/13_-phospholipase A2 signal transduction pathway, which promotes arachidonic acid release (Kurrasch-Orbaugh et al., 2003a,b).

Besides PLC-mediated Ca^2+^ signaling, 5-HT_2A_R activation also induces ERK phosphorylation via diverse intracellular signaling mechanisms (Gooz et al., 2006). Src and calmodulin promote 5-HT2AR-mediated phosphorylation of ERK. In the PC12 cell model system, ERK phosphorylation by 5-HT_2A_R may not depend on PLC/PKC signaling, and instead requires an increase in intracellular Ca^2+^, and the activation of CaM and Src (Quinn et al., 2002). The ERK target RSK2 directly acts on the third intracellular (i3) loop of 5-HT_2A_R protein (Sheﬄer et al., 2006), leading to direct phosphorylation of the i3 loop at the conserved residue Ser-314 to suppress 5-HT_2A_R signaling. In addition, RSK2 is required for tyrosine kinases, such as the epidermal growth factor receptor and the platelet-derived growth factor receptor, both of which have been demonstrated to attenuate 5-HT_2A_R functioning in primary cortical neurons (Strachan et al., 2009, 2010).

Besides the G protein, 5-HT_2A_Rs are also coupled to β-arrestin2. 5-HT binds 5-HT_2A_R to stimulate Akt phosphorylation via the β-arrestin2/phosphoinositide 3-kinase/Src/Akt cascade (Schmid and Bohn, 2010). Application of the 5-HT_2A_R agonist DOI to cultured cortical neurons induced phosphorylation of p21-activated kinase (PAK) via Rac guanine nucleotide exchange factor (RacGEF) kalirin-7 (Jones et al., 2009). The 5-HT_2A_R also regulates the tyrosine kinase pathway activity (Quinn et al., 2002). Excitation of neuronal 5-HT_2A_Rs activates transglutaminase which leads to transamidation of Rac1, a small G protein, resulting in constitutive activation of Rac1 (Dai et al., 2008). Chronic treatment with olanzapine, an atypical antipsychotic drug, causes the desensitization of 5-HT_2A_R signaling. In rat frontal cortex, stimulation of the JAK-STAT pathway desensitizes the 5-HT_2A_R-mediated PLC activation induced by olanzapine (Singh et al., 2010). Furthermore, constitutive activation of 5-HT_2A_Rs induces G_q/11_ phosphorylation and desensitization (uncoupling) (Shi et al., 2007).

As indicated above, 5-HT_2A_Rs are also expressed in microglia and mediate 5-HT-induced exosome release (Glebov et al., 2015). Activation of 5-HT_2A_R increases intracellular Ca^2+^ via PLC signaling in astrocytes (Hagberg et al., 1998) and Glu eﬄux from C6 glioma cells (Meller et al., 2002). Considering the diversity of signaling cascades that can be triggered by 5-HT_2A_R activation, it is perhaps not surprising that serotonergic activation of 5-HT_2A_Rs can have diverse influences on neuronal responses and CNS functions.

### Oligomerization

The GPCRs can form homomers and heteromers, and thereby present distinct signaling and functional activities (Rios et al., 2001). Consistent with this, 5-HT_2A_Rs have been shown to form oligomers (Lukasiewicz et al., 2010). Fluorescence resonance energy transfer and immunoprecipitation studies revealed that the human 5-HT_2A_R homodimerizes in cultured cells (Brea et al., 2009). For 5-HT_2A_R oligomers, the 5-HT_2A_R agonist DOI caused an increase in energy transfer efficiency to the level of 12%, and ketanserin caused a decrease of 4.4%. Heterodimers of 5-HT_2A_R and dopamine D_2_ receptors respond to DOI and quinpirole, a DA D_2_R agonist, with a decrease in FRET efficiency, while ketanserin and butaclamol increase the transfer efficiency value (Lukasiewicz et al., 2010). Heterodimers of 5-HT_2A_R and mGluR2 receptor form via the linking domain in transmembrane-4 and -5 segments, and are present in the human brain. Post-mortem studies indicate a reduced density of these functional complexes in brains of schizophrenics (Gonzalez-Maeso et al., 2008). Delta-9-tetrahydrocannabinol (THC), the main psychoactive compound of marijuana, induces memory impairments, anxiety, dependence, and analgesia. Vinals et al. (2015) recently reported that amnesic and anxiolytic effects, but not analgesia, induced by THC were suppressed in 5-HT_2A_R knockout mice. Molecular studies revealed that cannabinoid CB1 receptors (CB1R) and the 5-HT_2A_R physically interact with each other to form heteromers, which are distributed extensively in hippocampus, cortex, and dorsal striatum, but not in the NAc. *In vivo* experiments have revealed that stimulation of CB1R and 5-HT_2A_R reduces cell signaling, and the binding of an antagonist to one receptor blocks signaling of the interacting receptor. Heteromer formation leads to a switch in 5-HT_2A_R-mediated G-protein coupling from G_αq_ to G_i_. Synthetic peptides with the sequence of transmembrane helices 5 and 6 of CB1R disrupt CB1R and 5-HT_2A_R heteromerization *in vivo*, leading to a selective abrogation of memory impairments, but not the antinociceptive properties caused by THC exposure (Vinals et al., 2015). The anatomy, biology and function of 5-HT_2A_R homomers and heteromers, including the dynamic formation and dissociation, distribution, signaling and function, remain elusive. Elucidation of 5-HT_2A_R oligomers will be interesting for both basic science research and potential clinical applications.

### Allosteric Modulation

Recent years have witnessed a tremendous advance in the research and development of novel compounds for GPCRs that bind allosteric sites to regulate receptor structure and function. These ligands provide high specificity, novel modes of efficacy and may open up a novel avenue for therapeutic agents against multiple mental and neurological disorders. Allosteric modulators bind to a site distinct from that of the orthosteric ligand-binding site. Usually the allosteric modulator induces a structure change within the GPCR to enhance or suppress the orthosteric ligand’s functional activity (Conn et al., 2009; Melancon et al., 2012). Application of the amidated lipid, oleamide significantly potentiated 5-HT-induced hydrolysis of phosphoinositide in pituitary P11 cells expressing endogenously 5-HT_2A_Rs (Thomas et al., 1997). Taken together, these results indicate that there are several binding sites present on 5-HT_2A_Rs, and we suggest that it will be of interest to further characterize the functional significance of the distinct ligand-driven actions at the 5-HT_2A_R.

### Constitutive Activity

As mentioned above, 5-HT_2A_Rs can also be constitutively active (i.e., via activating the receptor in an agonist-independent activity) *in vivo* (Berg et al., 2008). The inverse 5-HT_2A_R agonists (e.g., risperidone and ketanserin) produce a great suppression of basal IP production, leading to a reduction of basal activity in the C322K mutant 5-HT_2A_R (Egan et al., 1998). The “constitutively active” arrestin mutant (Arr2-R169E) induces agonist-independent 5-HT_2A_R internalization, and a constitutive translocation of the Arr2-R169E mutant to the plasma membrane (Gray et al., 2003). The constitutive activity of 5-HT_2A_Rs may represent another mechanism of regulating cellular function. The specific relationships of these constitutively active 5-HT_2A_R-mediated properties to distinct behaviors have not been determined.

### Electrophysiological Characteristics

Electrophysiological studies reveal complex effects of 5-HT_2A_R activation on cortical neurons; however, mainly these receptors appear to mediate depolarizing effects on excitatory and inhibitory neurons. Slice recordings from prefrontal cortical neurons indicate depolarizing effects following 5-HT_2A_R activation (Aghajanian and Marek, 1999; Zhou and Hablitz, 1999; Avesar and Gulledge, 2012). Local application of DOI, a 5-HT_2A/2C_ receptor agonist, increases the firing rates of cortical neurons (Stein et al., 2000) and facilitates synaptic plasticity through an NMDAR-dependent mechanism in presumptive pyramidal neurons of the rat BLA (Chen et al., 2003). Meanwhile, α-methyl-5-hydroxytryptamine (a 5-HT_2_R agonist) and DOI induce activation of GABAergic interneurons of the rat BLA (Stein et al., 2000). Double immunofluorescence labeling demonstrated that the 5-HT_2A_R is primarily localized to parvalbumin-containing BLA interneurons. Accordingly, 5-HT primarily acts on 5-HT_2A_Rs to potentiate GABAergic inhibition. 5-HT_2A_R activation increases the frequency and amplitude of sIPSCs recorded from the pyramidal neurons in BLA of the juvenile rat (Jiang et al., 2009). DOI potentiates NMDAR-mediated changes in membrane potentials and calcium influx without affecting the neuronal resting membrane potential or input resistance. However, DOI does not affect AMPA/kainate receptor-mediated excitatory synaptic responses (Chen et al., 2003). The relationship of 5-HT_2A_Rs to NMDARs is consistent with the view that 5-HT_2A_Rs may be an effective target for modulating experience-dependent synaptic plasticity in the CNS. Globally, 5-HT_2A_Rs have been shown to influence low-frequency field potential oscillations in rat frontal cortex (Celada et al., 2008). Taken together, these findings demonstrate that the 5-HT_2A_R mediates 5-HT-induced excitation of cortical neurons. However, much remains to be determined as to the neurophysiological consequences of 5-HT_2A_R activation, in particular as they relate to the regulation of specific behaviors.

Recent molecular and pharmacological research has made significant advances in the understanding of the functional selectivity of 5-HT_2A_R. The multiple signaling pathways suggests bias agonism and bias signaling of 5-HT_2A_Rs, which posit that an agonist can produce a mix of signaling, which is potentially determined by cell type and functional status.

## 5-HT_2A_R Functions in CNS

Long-term declarative or episodic memory is supported by a network of brain structures in the medial temporal lobe of the mammalian brain. The medial temporal lobe memory system, which includes the hippocampus, dentate gyrus, and surrounding extrahippocampal cortical regions, influence decision-making processes guided by the PFC, and posterior parietal cortex (Squire et al., 2004, 2007; Preston and Eichenbaum, 2013). Serotonergic fibers originating from the raphe nuclei innervate many of the critical nodes within the medial temporal lobe memory system, including the hippocampus and amygdala, and on to the PFC (Vertes, 1991; Vertes et al., 1999). The modulatory influence of 5-HT on simple and more complex forms of learning and memory has been extensively examined in both invertebrate and vertebrate model systems (Kandel and Squire, 2000). The relevance of 5-HT to memory seems to generalize across mammals:, dietary tryptophan increases brain 5-HT levels and improves memory in rodents (Khaliq et al., 2006), the elderly, AD patients, and schizophrenics (Levkovitz et al., 2003; Porter et al., 2003). Further, reductions in brain 5-HT concentrations after acute or chronic tryptophan depletion has been demonstrated to impair contextual fear memory in mice (Uchida et al., 2007), object memory in rats (Jenkins et al., 2009), and declarative memory in humans (Schmitt et al., 2006). Below, we describe some evidence suggesting that the 5-HT_2A_R may hold special significance as one of the substrates by which 5-HT regulates learning and memory (Meneses, 2007).

### Learning and Memory

Polymorphisms in the human *HTR2A* gene are associated with altered memory processes. For example, a *HTR2A* gene polymorphism inducing the substitution of the His452 on the receptor subunit to a Tyr residue is associated with a significant impairment in memory recall amongst adults (de Quervain et al., 2003; Sigmund et al., 2008; Zhu et al., 2013). Carriers of the His452Tyr (rs6314) exhibited poor verbal delayed recall and recognition, but performed equivalent to controls on tests of immediate recall, attentional, and executive function (Wagner et al., 2008). Compared to His homozygotes, Tyr carriers exhibited a diminished hippocampal response to novel stimuli and a higher tendency to judge novel stimuli as familiar during delayed recognition (Schott et al., 2011). Amongst schizophrenics and healthy controls, those carriers of homozygous CC (T102C) and GG (A-1438G), or carriers of the so-called *T*-allele (rs6314), of the *HTR2A* gene polymorphisms exhibited significantly impaired short-term verbal memory (Alfimova et al., 2009), and spatial working memory (Blasi et al., 2013). Another polymorphism in the *HTR2A* gene, referred to as rs4941573 was found to be predictive of increased error rate in a spatial working memory task in an adult Chinese subject population (Gong et al., 2011). These results provide just a brief and incomplete view of a broad literature indicating the impressive degree to which alterations in the *HTR2A* gene relate to disordered cognitive functions in normal and abnormal human subjects.

The regional distribution of 5-HT_2A_Rs can be predictive of the memory capacities that are sensitive to serotonin manipulation. The 5-HT_2A_Rs are widely expressed in the neocortex and hippocampus of rats (Xu and Pandey, 2000; Hannon and Hoyer, 2008), rabbits (Aloyo and Harvey, 2000), primates (Jakab and Goldman-Rakic, 1998; Lopez-Gimenez et al., 1998), and humans (Hoyer et al., 1986; Lopez-Gimenez et al., 1998). **Table 1** summarizes the major findings of studies in which the learning and memory effects were examined after 5-HT_2A_R pharmacological manipulations across distinct tasks and different species. The inconsistency of experimental results may be attributed to the species, selectivity and dose of drug, behavioral task and other effectors.

**Table 1:** Reported effects on learning and memory after pharmacological manipulation of 5-HT_2A_ receptors (5-HT_2A_Rs).

Drug	Route; dose	Task	Species	Effect	Reference
M100907	0.01–0.1 mg/kg; i.p.	Probabilistic reversal learning	Mice	↑Acquisition	Amodeo et al., 2014; ^∗^BTBR T+tf/J mouse model of autism
M100907	0.01–0.1 mg/kg; i.p.	Serial spatial reversal learning task	Rats	↓ Retrieval	Boulougouris et al., 2008
M100907	0.02–2.0 nmol; olfactory bulb	Reversal-learning task	Rats	↓ Acquisition	Furr et al., 2012
MDL 11,939	0.067–6.7 μmol/kg; s.c.	Nictitating membrane conditioned responses	Rabbits	↓ Acquisition	Welsh et al., 1998
MDL 11,939	300 ng/μl; mPFC	NOR task	Rats	↓ Retrieval	Bekinschtein et al., 2013
Ritanserin,	2.5 mg/kg × 11 days; s.c.	Conditioned olfactory training	Rat pup	↑ Acquisition	McLean et al., 1996
Risperidone	1 mg/kg; i.p.	Reward-dependent operant conditioning task	Rats	↓ Acquisition and ↑ extinction	Frick et al., 2015
Risperidone	0.125 mg; i.p.	Probabilistic reversal learning	B6 mice	↓ Acquisition	Amodeo et al., 2014
Ketanserin Methysergide	1.0–3.0 mg/kg; s.c.; 3.0–15.0 mg/kg; i.p.	Delayed non-matching to position task (working memory)	Rats	↔ Retrieval	Ruotsalainen et al., 1997
Ketanserin	0.1 mg/kg × 14 days; i.p.	Passive avoidance paradigm and MWM	Rats	↓ Acquisition	Fedotova and Ordyan, 2010
DOI Ketanserin	0.01–0.1 mg/kg; i.p. 0.001–0.1 mg/kg, i.p.	Autoshaping learning task	Rats	↑ Consolidation	Meneses et al., 1997
M100907; α-methyl-5-HT	PFC	Oculomotor delayed-response tasks	Monkeys	↓ Acquisition ↑ Acquisition	Williams et al., 2002
TCB-2	1.0 mg/kg; i.p.	NOR task and Trace and delay fear conditioning	Mice	↑ Object memory acquisition; ↑fear memory extinction	Zhang et al., 2013
DOI	0.1–0.3 mg/kg; i.p.	Autoshaping learning task	Rats	↓ Consolidation	Meneses, 2007
LSD	0.43–12.9 μg/side; hippocampus	Trace eyeblink conditioning.	Rabbits	↑ Acquisition	Romano et al., 2010
LSD	1–300 nmol/kg; i.v.	Nictitating membrane response	Rabbit	↑ Acquisition	Gimpl et al., 1979
LSD	0.13 mg/kg/d × 11 days; s.c.	Bulbectomy-induced deficit in active avoidance learning	Rats	↑ Acquisition	Buchborn et al., 2014
Psilocybin	215 μg/kg; oral	Spatial working memory task	Humans	↔ Retrieval	Carter et al., 2005
Psilocybin	0.1–1.5 mg/kg, i.p.	Trace fear conditioning -	mice	↑ Extinction	Catlow et al., 2013
Psilocin	1.0 mg/kg, i.p. 4.0 mg/kg, i.p.	MWM; Carousel maze (CM)	Rats	↓Acquisition of CM; ↓Retrieval of MWM (4 mg/kg); ↔ Consolidation	Rambousek et al., 2014
Quipazine	1.25–10 mg/kg, s.c.	Conditioned avoidance response	Rats	↑ Acquisition	Alhaider et al., 1993

*↑, enhance; ↓, suppress; ↔, no effect.*

#### Object Memory

**FIGURE 1:**
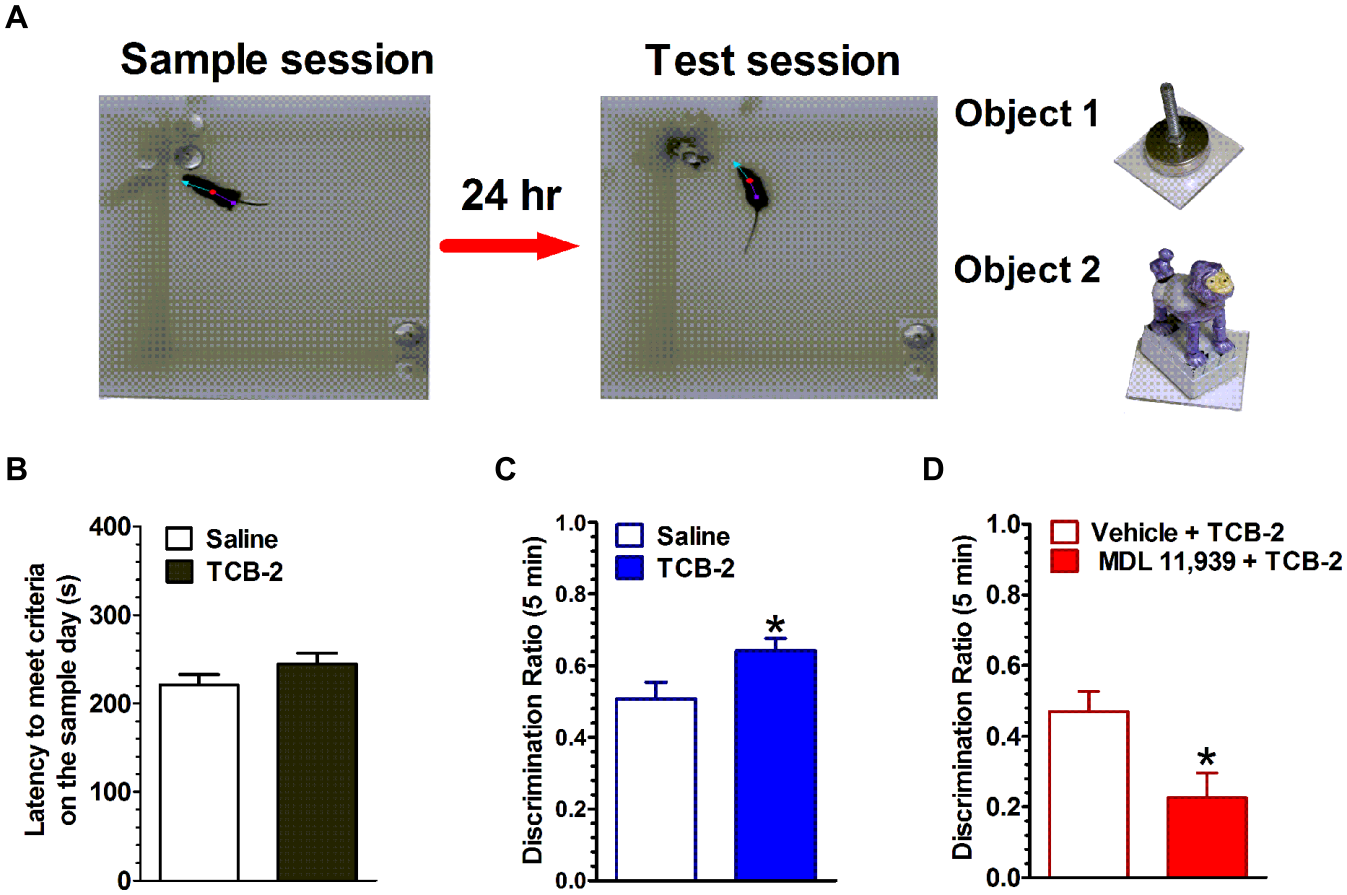
**Activation of 5-HT_2A_Rs enhances the consolidation of object memory. (A)** Experimental strategy. Left, during the sample session, mice were allowed to explore two identical novel objects each for at least 15 s, or either one for 18 s within a 10-min sample session. Middle, during the test session 24 h later, one of the objects (a cabinet leveling foot) was replaced with a novel object (a plastic toy monkey) and mice were individually reintroduced to the arena. Right, the objects used in this study. **(B)** There was no difference in the latency to achieve exploration criteria during the sample session between the saline- and TCB-2-treated mice. **(C)** Mice that received TCB-2 right after the sample session exhibited a significantly stronger preference for exploring the novel object during the test session: analysis revealed that TCB-2-treated mice had a higher mean discrimination ratio as compared to that of the saline group. **(D)** MDL 11,939, a selective 5-HT_2A_R blocker suppressed TCB-2-induced enhancement in object memory (Zhang et al., 2013). ^∗^*p* < 0.05, vs. respective control condition.

The spontaneous NOR task, which relies on rodents’ inherent preference for exploring novel over familiar stimuli, has become a popular method for examining the neuropharmacological and neurophysiological mechanisms of object memory (Ennaceur, 2010; Cohen and Stackman, 2015). In the task, rodents are exposed to one or two novel objects in a familiar enclosure during a sample session (i.e., training). The rodent is removed from the enclosure after it has sufficiently explored the objects. After a delay of some length, the rodent is returned to the enclosure for a memory test session, during which the enclosure contains one familiar object and a novel object. If the rodent has successfully encoded and consolidated the memory of the original object from the sample session, then it is expected that the rodent will preferentially explore the novel object during the test session. The NOR task offers advantages for testing rodent memory in that the distinct memory processes of encoding, consolidation and retrieval are operationally defined as events occurring during the sample session, after the sample session, or during the test session, respectively. Another advantage is that the behavioral responses are spontaneous rather than requiring overt motivation such as electrical shock or food restriction. Our recent studies implicate the hippocampus as a key region in the rodent brain for object memory processes (Cohen et al., 2013; Cohen and Stackman, 2015). In light of the fact that 5-HT_2A_Rs are densely expressed in the hippocampus (Luttgen et al., 2004), we examined the contribution of hippocampal 5-HT_2A_Rs in object memory processes in male mice using an NOR task (see **Figure 1**). Systemic activation of 5-HT_2A_Rs with the selective agonist, TCB-2 after the sample session significantly enhanced the time mice spent exploring the new object presented during the test session 24 h later (Zhang et al., 2013). The memory-enhancing effect of TCB-2, was blocked by pretreatment with the 5-HT_2A_R antagonist, MDL 11,939, which suggests that 5-HT_2A_R activation enhances the consolidation of object memory. Interestingly, when TCB-2 was administrated before the sample session, or before the test session, the 5-HT_2A_R agonist failed to increase novel object preference relative to the respective control group. Together, these data suggest that 5-HT_2A_R activation selectively potentiates memory consolidation. Furthermore, the selective local microinfusion of TCB-2 into the CA1 region of dorsal hippocampus recapitulated the memory enhancing effect observed after systemic treatment (Zhang et al., 2015). The relevance of the 5-HT_2A_R for object memory processes was also demonstrated by results of a study showing that the local infusion of the 5-HT_2A_R antagonist MDL 11,939 into the mPFC impaired retrieval of object-in-context memory in rats (Bekinschtein et al., 2013). Interestingly, the 5-HT_2A_R agonist DOI was found to impair retrieval of memory for an operant response by adult rats in an autoshaping task (Meneses, 2007). Thus, it would appear that the influence of the 5-HT_2A_R on memory is task- and memory system-dependent, and perhaps by the underlying neural circuitry that supports the respective memory process.

The encoding and consolidation of hippocampal-dependent memory appears, in part, to require fast glutamatergic neurotransmission, ensuing phases of synaptic plasticity, and dynamic replay of experience-dependent neurophysiological oscillatory activity within hippocampal cell populations (Eichenbaum, 1999; Karlsson and Frank, 2009). Our published (Zhang et al., 2013) data show that post-training activation of 5-HT_2A_R s enhances object memory, likely by affecting consolidation. Prevailing views state that the hippocampus transfers recent to-be-remembered information to the neocortex during sharp wave ripples of the hippocampal local field potential (i.e., 100–200 Hz ripples; Chrobak and Buzsaki, 1996; Carr et al., 2011). During sleep, hippocampal neurons ‘replay’ patterns of spike trains present during a learning episode. As sharp wave ripples and replay may represent systems consolidation of memory, it would be of interest to examine the influence of 5-HT_2A_R-sensitive drugs on sharp wave ripples and replay of spiking sequences during sleep episodes after a to-be-remembered experience. Postsynaptic 5-HT_2A_Rs may modulate object memory consolidation by also influencing NMDAR-mediated synaptic plasticity. Consistent with this possibility, hippocampal 5-HT_2A_Rs are predominantly expressed at dendritic sites on pyramidal neurons (Cornea-Hebert et al., 1999; Peddie et al., 2008). 5-HT_2A_R-containing dendritic processes also were immunolabeled for the NMDAR subunit NR1 and GluR2 (Peddie et al., 2008). We have found that 5-HT_2A_R activation increased the extracellular eﬄux of glutamate in the dorsal hippocampus, and increased the basal firing rates of CA1 pyramidal neurons in awake behaving mice (Zhang et al., 2015). These results suggest that the 5-HT_2A_R activation induced facilitation of object memory consolidation, may result from the potentiation of hippocampal glutamate release, and pyramidal neuron temporal dynamics at a critical post-training time period. These data suggest that the 5HT_2A_R may serve as a drug target for pharmacological interventions to treat memory impairments. It is conceivable that 5-HT_2A_R activation promotes an increase in intracellular Ca^2+^, combined with NMDAR-mediated Ca^2+^ influx, which together would facilitate the behavior-initiated synaptic plasticity. Aghajanian and Marek (1999) reported that activation of 5-HT_2A_R produces an elevation in the frequency and amplitude of neuronal sEPSP/sEPSC. Consistently, 5-HT_2A_R activation has been shown to facilitate NMDAR activity and synaptic plasticity in the cortex (Arvanov et al., 1999) and BLA (Chen et al., 2003). Furthermore, 5-HT_2A_R directly interacts with PSD-95 to regulate receptor trafficking and signaling (Xia et al., 2003). 5-HT_2A_R activation induces a transient increase in dendritic spinogenesis (Yoshida et al., 2011), phosphorylation of PAK, neuronal Rac guanine nucleotide exchange factor (Jones et al., 2009), BDNF expression (Vaidya et al., 1997), and Erk mitogen-activated protein kinase activity (Florian and Watts, 1998; Watts, 1998). Finally, the 5-HT_2A_R inverse agonist pimavanserin was shown to reverse NMDAR antagonism-induced object memory impairments in combination with atypical antipsychotic drugs (Snigdha et al., 2010). These results support the view of a modulatory influence of 5-HT_2A_R on NMDAR-dependent memory mechanisms. Considering the myriad potential influences of 5-HT_2A_R on medial temporal lobe memory mechanisms, there would appear to be multiple downstream influences by which 5-HT_2A_R activation could enhance memory.

#### Fear Memory

While there is a rich literature on the influence of serotonin on anxiety and an established contribution of serotonergic drugs to the remediation of anxiety disorders in humans, the present review will focus on the influence of 5-HT_2A_Rs on fear memory encoded during Pavlovian conditioning sessions. Pavlovian fear conditioning has become a popular procedure for examining the neurobiological mechanisms of fear memory. As a Pavlovian conditioning procedure, fear conditioning lends itself well to defining processes of encoding, consolidation and retrieval of fear memory. Fear conditioning taxes a well-defined neural circuit within the amygdala, which in turn interacts with the hippocampus, anterior cingulate, or the PFC, depending on the elements of the conditioning session and the stage of memory processing (Zelikowsky et al., 2014). In addition, considerable attention has been given to investigations of the underlying biology of extinction of fear memory. During a delay fear conditioning session, an innocuous stimulus (e.g., a neutral tone or light) becomes a CS when it is repeatedly presented in such a way that it co-terminates with the presentation of a sufficiently aversive US (e.g., foot shock) (Zhang et al., 2013). The unconditioned response to the foot shock US is typically jumping and running, but the conditioned response to the CS is a defensive freezing response, or the cessation of all movement except for respiration. Thus, the freezing behavior provides a reliable post-conditioning measure of fear memory in rodents (Blanchard and Blanchard, 1969). During fear conditioning, the subject learns to associate the tone CS with the foot shock US, and under certain conditions, learns to associate the foot shock with the environment or context where the conditioning session was presented. Acquisition of both the tone-shock and the context-shock associations requires the amygdala; however, the context-shock associations are also dependent upon the hippocampus (Kim and Fanselow, 1992; Phillips and LeDoux, 1992). There has been considerable debate regarding the involvement of the hippocampus in contextual fear memory since there have been reports that hippocampal lesions impair contextual fear memory (Kim and Fanselow, 1992; Phillips and LeDoux, 1992; Anagnostaras et al., 1999; Stiedl et al., 2000), and others reporting that such lesions spare contextual fear memory (Cho et al., 1999; Wiltgen et al., 2006). Consensus seems to be building for the view that if the rodent is permitted sufficient time to acquire a hippocampal-dependent configural representation of the context (the chamber’s geometry, olfactory, visual, tactile, and auditory cues) before the US is presented, then the hippocampus is engaged in associating the contextual memory with the foot shock (Rudy et al., 2002, 2004; Matus-Amat et al., 2004; Zelikowsky et al., 2014). In a trace fear conditioning procedure, a temporal gap is imposed between the offset of the tone CS and the onset of the foot shock US. The acquisition of an appropriately timed (i.e., anticipatory) conditioned freezing response occurs progressively over the course of the repeated CS–US pairings; this temporal fear memory is a form of declarative memory dependent on intact hippocampal function in rodents and humans (Clark and Squire, 1998; McEchron et al., 1998; Chowdhury et al., 2005). It should be clear that in deciphering an influence of 5-HT_2A_R-sensitive drugs on the distinct processes of memory for contextual and/or cued fear, one must consider the specifics of the conditioning protocol used.

Finally, considerable attention has been directed toward defining the mechanisms of fear memory extinction, in part because of extinction’s potential relationship to components of the human disorder post-traumatic stress disorder (Jovanovic and Ressler, 2010). Repeated presentations of the CS alone to the fear-conditioned rodent, promotes the acquisition of a new inhibitory association, which dampens or completely suppresses the expression of conditioned fear responses. Distinct subregions of the rodent PFC contribute differentially to fear extinction; that is, the prelimbic cortex appears to influence the expression of fear responses, while the infralimbic cortex influences the acquisition of extinction of fear memory (Quirk et al., 2010; Sierra-Mercado et al., 2011). Synaptic plasticity within mPFC-BLA neuronal circuits is induced during fear extinction training, resulting in increased inhibition of CS-elicited activity of BLA extinction neurons (Herry et al., 2008, 2010). Thus, converging evidence implicates the infralimbic and prelimbic cortices of the rodent brain and their differential projections to the amygdala sub-regions and to the hippocampus as contributing significantly to the synaptic plasticity that develops during the acquisition of fear extinction (see Tovote et al., 2015 for a recent review).

**FIGURE 2:**
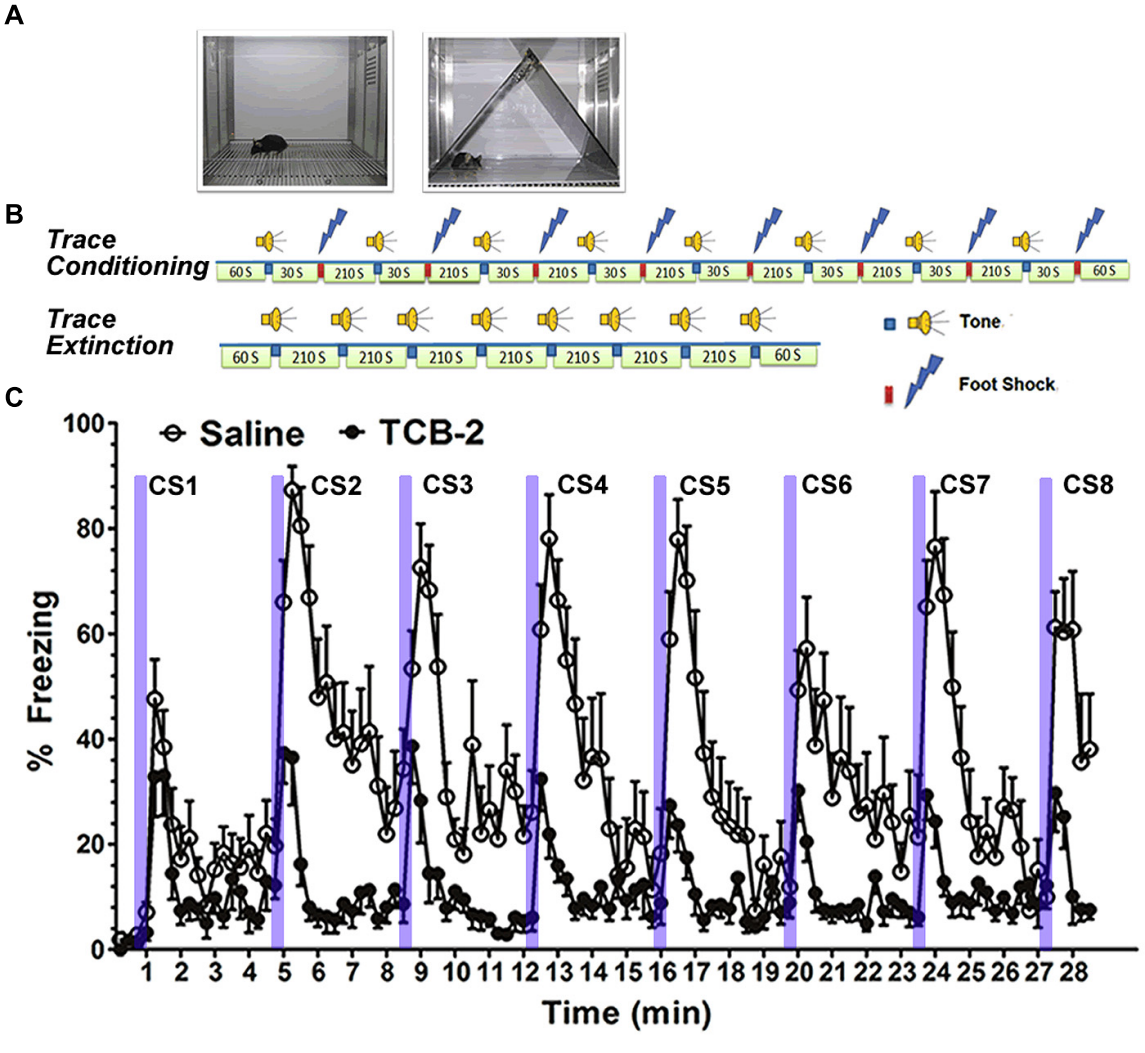
**Stimulation of 5-HT_2A_Rs enhances the acquisition of extinction of trace fear memory. (A)** Left, a chamber for fear conditioning (Context A) and contextual fear memory test; right, Context B, a modified chamber with different color, context, light density, and odor for cued fear memory test. **(B)** Trace fear conditioning training procedure. After a 60-s habitation to Context A, a tone was presented for 15 s followed by 30 s stimulus-free interval, and then a 0.5-s, 0.75 mA foot shock (US) was presented. The CS–US pairing was repeated eight times with a 210-s intertrial interval (ITI). Mice were removed from the conditioning chamber and returned to their home cages 60 s after the final CS–US pairing. Trace fear conditioning extinction procedure. Sixty seconds after placing the mouse into the modified chamber, eight unpaired 15-s tone CS were presented with a 120-s ITI. **(C)** Mice that received TCB-2 (1.0 mg/kg, i.p.) before a trace fear memory extinction test exhibited accelerated acquisition of extinction as indicated by significantly lower freezing scores earlier in the course of extinction as compared to those of vehicle-treated mice. TCB-2 significantly decreased percent freezing from the second to eighth CS presentation (Zhang et al., 2013).

We found that systemic administration of the 5-HT_2A_R agonist TCB-2 (see **Figure 2**) significantly enhanced the acquisition of fear extinction in mice that had undergone trace fear conditioning or delay fear conditioning (Zhang et al., 2013). Importantly, the 5-HT_2A_R agonist did not affect locomotor responses or baseline freezing in the mice. Therefore, the effect of TCB-2 on fear extinction appeared to be specific to facilitating the acquisition of the new inhibitory memory that suppressed fear expression. It is of interest to determine the site of action in the rodent brain at which TCB-2 works to facilitate fear extinction. In light of the plastic changes in neural circuitry that occur during the acquisition of fear extinction, it is possible that TCB-2 influences either the infralimbic cortical neurons or the “extinction neurons” of the BLA to facilitate fear extinction. Izumi and colleagues reported that an amygdala-selective reduction of 5-HT content via site-specific 5,7-DHT injection reduced the expression of conditioned fear responses in rats (Izumi et al., 2012). While this finding is difficult to reconcile with our report that 5-HT_2A_R activation enhanced fear extinction, it is possible that the 5-HT denervation may have increased postsynaptic expression of 5-HT_2A_Rs in the amygdala, which might in turn impair the expression of fear. It is clear that further studies are needed to clarify the neurophysiological influences of 5-HT, and the 5-HT_2A_R in particular, on the neural circuitry supporting fear memory encoding, consolidation, retrieval, and extinction.

The influence of the 5-HT_2A_R on the extinction and reconsolidation of fear memory may have significant impact on the development of therapeutic approaches for subjects with fear memory invasion, such as phobias and post trauma stress disorder (Quirk et al., 2010). For decades, the pharmacological manipulation of the 5-HT system has been a useful approach to treat emotional and mental disorders, such as depression and anxiety. Recent progress has suggested a promising therapeutic application of hallucinogenic 5-HT_2_ agonists to treat depression and anxiety (Grob et al., 2011). These results suggest that despite the historical stigma associated with 5-HT_2A_R activators as potential hallucinogens, such compounds may provide important medical potential for treating affective and cognitive symptoms associated with emotional and mental conditions.

Glutamatergic neurons in the amygdala, cortex and hippocampus are essential for memory extinction and reconsolidation. Local infusion of NMDAR antagonists into the BLA or CA1 region of hippocampus before extinction training suppresses fear memory extinction and reconsolidation (Baker and Azorlosa, 1996; Szapiro et al., 2003). The NMDAR partial agonist D-cycloserine facilitates the extinction of fear memory (Walker et al., 2002; Ledgerwood et al., 2003). Knockout of NMDAR in hippocampal CA1 pyramidal cells exclusively impairs the establishment of conditioning between the CS and the US during a trace fear conditioning task. These results suggest that the CS representation and conditioning are entrained within hippocampus cell ensembles, probably via NMDAR-dependent synaptic plasticity (McHugh et al., 1996; Huerta et al., 2000). Recall that 5-HT_2A_Rs are expressed in the dendrites and dendritic spines of dentate gyrus neurons where NMDARs and AMPARs are assumed to be located (Peddie et al., 2008). 5-HT_2A_R activation produces an elevation in the frequency and amplitude of cortical neuronal sEPSP/sEPSCs (Aghajanian and Marek, 1999), facilitates NMDAR activity and synaptic plasticity in the cortex (Arvanov et al., 1999) and BLA (Chen et al., 2003). It is worth while to examine the degree to which NMDARs expressed in the infralimbic and prelimbic cortices contribute to the 5HT_2A_R-mediated enhancement in fear extinction.

Converging evidence demonstrates that activation of 5-HT_2A_Rs via systemic injection, or by local microinfusion, appears to enhance two forms of hippocampal-dependent memory in mice: object memory and conditioned fear memory. Administration of a selective 5-HT_2A_R antagonist alone was not found to significantly affect object memory or fear memory (Zhang et al., 2013), suggesting that memory consolidation does not require serotonergic activation of 5-HT_2A_Rs and/or the antagonists do not affect the tonic effect the 5-HT_2A_R. Activation of 5-HT_2A_Rs with TCB-2 was also found to facilitate fear memory extinction in mice. These results offer promising support for the view that the 5-HT_2A_R may be an important new target for consideration in the search for mechanisms by which long-term memory can be enhanced in humans.

### Hallucination vs. Spatial Cognition

#### 5-HT_2A_R and Hallucination

Recent evidence suggests that activation of 5-HT_2A_Rs may promote experiencing visual hallucinations by increasing neuronal excitability and altering visual-evoked cortical responses (Kometer et al., 2013). Hallucination is a type of misperception defined as the perception of an object without there being an object to perceive. Hallucinations are a significant characteristic found in a diversity of psychiatric and neurological states. Hallucinations can be triggered by at least three categories of drugs: psychedelics, (i.e., DOI, TCB-2, LSD, and psilocybin) via activation of 5-HT_2A_Rs, psychostimulants (i.e., cocaine or amphetamine) via activation of dopamine D2 receptors and dissociative anesthetics (i.e., phencyclidine or ketamine) via blockade of glutamate NMDARs. The signaling and behavioral responses to each hallucinogen are distinct from each other. Activation of 5-HT_2A_R is critical for the psilocybin (found in magic mushroom)-induced α oscillations, N170 visual-evoked potentials, and visual hallucinations (Kometer et al., 2013).

5-hydroxytryptamine/serotonin is an endogenous neurotransmitter and is not considered hallucinogenic. Interesting, *N*-methyltryptamines, a metabolite of 5-HT, also presents high affinity for 5-HT_2A_R and can induce hallucinations in a manner independent of β-arrestin2/phosphoinositide 3-kinase/Src/Akt cascade (Schmid and Bohn, 2010). Signaling for hallucinogens is distinct. Lisuride (an antiparkinsonian agent) and LSD both bind cortical 5-HT_2A_R, and thereby regulate PLC activity. LSD signaling involves pertussis toxin-sensitive heterotrimeric G_i/o_ proteins and Src (Gonzalez-Maeso et al., 2007). Non-hallucinogenic agonists, for example lisuride, only stimulate cortical Gq in rats, whereas hallucinogens such as psilocybin (found in magic mushrooms), and LSD stimulate both G_q/11_ and G_i_ (Gonzalez-Maeso et al., 2007). The β-arrestin pathway is involved in hallucinogen-mediated head shake responses in rodents (Schmid et al., 2008), and 5-HT induces a head shake response in mice via a β-arrestin-2-dependent signaling. However, the DOI invoked head shake behavior is not dependent upon β-arrestin-2 signaling. These findings suggest that the 5-HT_2A_R-β-arrestin interaction may be exclusively for endogenous 5-HT action. Further examination of hallucinogen-mediated signaling may have major implications in drug development for treating emotional and mental disorders such as depression and schizophrenia (Schmid et al., 2008). More research efforts will need to be focused on the hallucination-inducing aspects of 5-HT_2A_R-sensitive drugs and, relevant to their potential therapeutic potential, it may be important to consider designing novel compounds that yield more of the beneficial effects, without activating those problematic sensory and perceptual effects.

#### 5-HT_2A_R-mediated Hallucination and Spatial Cognition

5-HT_2A_ receptors may affect spatial cognition. A human population-based study shows that 5-HT_2A_R TT genotype of rs6313 is associated with better spatial cognitive performance (Gong et al., 2011). Kant et al. (1998) reported that the 5-HT_2A_R agonist DOI (0.1 and 0.25 mg/kg, 30 min pretreatment) slowed rat performance as assessed by swim time on both a well-learned water maze as well as learning of a new maze, but DOI did not alter error rate on either task. Kant concluded that DOI impaired performance by suppressing motor activity on a water maze (Kant et al., 1998), which was in opposition to another report showing that manipulation of 5-HT_2A_R did not impair the latency to a visible platform water maze test (Naghdi and Harooni, 2005). The serotonergic hallucinogens may impair the hippocampal-dependent spatial cognition by acting on 5-HT_2A_Rs (Naghdi and Harooni, 2005). However, the direct evidence of 5-HT_2A_R on visuospatial cognition and the central target has not been determined.

Serotonergic psychedelics may affect the integrity of visual functioning. Visual-directed spatial cognition and navigation are guided by exteroceptive (e.g., landmarks) and interoceptive (e.g., self-motion information) cues, and their integration. The hippocampus is a pivotal brain region receiving and integrating information for spatial memory and navigation in rodents (Broadbent et al., 2004; Eichenbaum, 2004). The MWM is a classic behavioral task for testing hippocampal-dependent visuospatial cognition, including place learning and memory, orientation and decision-making (Morris et al., 1982; Morris, 1984). Further, hippocampal place cells exhibit location-specific firing, and are considered to be fundamental components of network for spatial problem solving in the mammalian brain (for a review see Moser et al., 2008). The hippocampal neural circuit representing current location, directional heading and its integration is influenced by exteroceptive and interoceptive cues, and is considered to guide spatial cognition and navigation.

We recently found that pre-test activation of 5-HT_2A_R with TCB-2 significantly delayed the initiation of an accurate search path by well-trained male mice in the hidden platform MWM (Zhang et al., 2015). Importantly, 5-HT_2A_R activation did not affect swim performance or visual cue-triggered approach behavior in the visible platform water maze task. Taken together, our results suggest that the activation of 5-HT_2A_R impairs the retrieval of hippocampal spatial memory, but not the accuracy of spatial information retrieval and decision-making. It is conceivable that the delayed initiation of accurate spatial search by TCB-2-treated mice might reflect the possible visual hallucinatory influences of the 5-HT_2A_R agonist. For example, perhaps TCB-2-induced a brief aberration of visual input that slowed the perception of current position and local view of the mouse at the start of the water maze probe test. Once, reconciled or reoriented, the mouse was able to swim accurately to the remembered spatial location of the platform. It will be of interest to determine where in the brain TCB-2 is acting to alter spatial memory retrieval. The relatively weak influence of TCB-2-induced visual hallucination on spatial navigation may due to the difference in the visual information passing through the brain and central targets processing the information.

Taken together, the results we have reported here of memory effects after activation of the 5-HT_2A_R represent a fairly complex picture. The post-training administration of TCB-2 enhanced consolidation of object memory in mice. Pre-test administration of TCB-2 did not affect retrieval of object memory, yet delayed retrieval of spatial memory. Pre-extinction training administration of TCB-2 facilitated the acquisition of extinction of both trace and delay fear memories. The facilitating effect of TCB-2 on fear extinction may have been the result of a combined effect of suppressing fear expression – possibly a consequence of impaired retrieval of fear memory, and enhancing the encoding and consolidation of fear extinction. To characterize the 5-HT_2A_R agonist as a cognitive enhancer based solely on our object memory results, would be to ignore the other experimental findings. We are interested in conducting a more comprehensive analysis of the impact of TCB-2 on multiple forms of memory. For example, it will be interesting to examine whether post-conditioning TCB-2 might enhance the consolidation of fear memory, in a manner consistent with that observed in the NOR task. Likewise, it will be interesting to test whether post-extinction training TCB-2 facilitates the consolidation of fear extinction. Results of these experiments will help in better appreciating the modulatory influence of the 5-HT_2A_R on long-term memory processes. This synthesis of recent findings of the influences of 5-HT_2A_R activation should provide a credible argument that the 5-HT_2A_R participates significantly to the well-documented contribution of 5-HT to memory (Meneses, 2013).

### 5-HT_2A_R and Mental Disorders

A number of psychiatric and neurodegenerative disorders are associated with the variation of structure, expression, and function of 5-HT_2A_Rs. Positron emission tomography (PET) molecular imaging has the sensitivity to quantify binding of 5-HT_2A_Rs in CNS disorders. Medication-free depressed subjects presented greater 5-HT_2A_R binding (Bhagwagar et al., 2006). There was a significant reduction in 5-HT_2A_R binding in frontal polar, dorsolateral and medial frontal cortex, and parietal and temporal associative cortex of OCD patients and a significant correlation between 5-HT_2A_R availability in orbitofrontal and dorsolateral frontal cortex and clinical severity (Perani et al., 2008). Schizophrenia patients present with very high 5HT_2A_R occupancy in the frontal cortex (Talvik-Lotfi et al., 2000). These results suggest that the variation in the number, affinity and/or function of 5-HT_2A_R participates in the etiology of mental disorders.

#### Alzheimer’s Disease

It is interesting to note that neocortical 5-HT_2A_R binding is significantly decreased in patients with early stage AD, and in those with mild cognitive impairment; especially in temporal lobe regions associated with long-term memory (Meltzer et al., 1998; Hasselbalch et al., 2008; Santhosh et al., 2009; Marner et al., 2011, 2012). Further, the severity of cognitive impairment in AD patients correlates with the decrease in 5-HT_2A_R binding (Versijpt et al., 2003). Given the pattern of 5-HT_2A_R distribution in neocortical regions and their expression on principal excitatory neurons, it is possible that the marked reduction in 5-HT_2A_R in brains of AD is a direct product of neuron loss in key brain regions. Consistent with evidence from the human studies, the Alzheimer’s-like neuropathology and associated memory deficits in rodents, which follow intra-hippocampal injection of ß-amyloid(1-42), are associated with a significant reduction in levels of hippocampal 5-HT_2A_R expression (Christensen et al., 2008). Although we have focused this analysis on the influence of 5-HT_2A_Rs on long-term, hippocampal-dependent memory, there is clear and compelling evidence to suggest that the 5-HT_2A_R represents a potential new target by which human long-term memory may be modulated. We assert that it will be of interest in further examine the contribution of 5-HT_2A_Rs to memory processes, and we are particularly interested in determining neurophysiological influences of 5-HT_2A_R agonists which promote the enhancement of memory consolidation which we have reported in mice.

#### Drug Memory

Drug dependence, classified as an impulsive, compulsive, and relapsing psychiatric disorder, represents a devastating societal problem worldwide. The profound symptoms of drug abuse, in particular the cue-elicited relapse to drug use after even long periods of abstinence, are a consequence of robust experience-dependent synaptic plasticity within the brain’s reward circuit. Like episodic, semantic, and habit memory, drug-associated memories are persistent and hold a strong influence on current and future behaviors. Of particular interest is the consideration of memory extinction as a psychological tool for remediating the problem of relapse in drug addicts. That is, if the problem of drug abuse is approached as a mental disorder of memory, then pharmacological manipulations that facilitate extinction may hold therapeutic utility for treating drug abuse. Drug exposure alters the expression and function of 5-HT_2A_R, for example morphine decreases frontocortical 5-HT_2A_R binding affinity in dogs (Adriaens et al., 2012). 5-HT_2_Rs are up-regulated in amygdala, midbrain, pons, and medulla of morphine-tolerant and -dependent rats, but not in morphine-abstinent rats (Gulati and Bhargava, 1989). There is considerable evidence that 5-HT_2A_Rs modulate the behavioral consequences of repeated exposure to addictive psychomotor stimulants. For example, M100907 suppresses hyperactivity elicited by cocaine (Fletcher et al., 2002), MK-801, amphetamine (O’Neill et al., 1999), and morphine (Auclair et al., 2004). DOM, a 5-HT_2A_R agonist, attenuates locomotor-stimulating effects of morphine, which could be prevented by M100907 (Li et al., 2013). Furthermore, M100907 attenuated the ability of experimenter-administered cocaine to reinstate lever pressing (Fletcher et al., 2002) and attenuated the drug associated cue-induced reinstatement of cocaine-seeking behavior after extinction (Nic Dhonnchadha et al., 2009). M100907 also suppressed reinstatement induced by nicotine prime or nicotine-associated cue (Fletcher et al., 2012) and sensitization (Zaniewska et al., 2010). Intra-NAc infusions of M 100907 blocked the expression of cocaine-induced locomotor sensitization (Zayara et al., 2011). Intra-PFC M100907 decreased cue-elicited reinstatement of cocaine seeking-behavior (Pockros et al., 2011). Together, these results suggest that 5-HT_2A_Rs modulate drug addiction-dependent behaviors such as craving and drug-seeking and pharmacological blockade of 5-HT_2A_Rs may represent a therapeutic advance in suppression of cue-evoked craving and/or relapse in drug addicts.

#### Therapeutic Application of 5-HT_2A_R

Preclinical and clinical studies have provided support for the use of pharmacological manipulation of 5-HT_2A_R to treat the symptoms of mental disorders. Activation of 5-HT_2A_R with TCB-2 in the medial septum-diagonal band of Broca complex enhances neuronal activity and working memory in hemiparkinsonian rats (Li et al., 2015). M100907 had no effect on attentional performance, but abolished the PCP-induced attentional performance deficits in rats (Poyurovsky et al., 2003). M100907 prevents impairment in attentional performance by NMDAR blockade in the rat PFC (Mirjana et al., 2004). There are a number of 5-HT_2A_R drugs that have been evaluated or are being currently evaluated under clinical trials, for example quetiapine^1^ for schizophrenia; M100907^2^ for depression; ACP-103^3^ for Parkinson’s disease; pimavanserin for patients with AD psychosis^4^ or with Parkinson’s disease psychosis^5^.

## Conclusion

In this review, we have summarized recent progress in the signaling, polymerization and allosteric modulation of 5-HT_2A_R; and have discussed the critical role of 5-HT_2A_Rs in a number of cognitive processes. Based on the results of studies from our lab and others, it appears that activation of 5-HT_2A_Rs may offer a novel approach to treat the impairment of learning and memory associated with several neurodegenerative disorders. Meanwhile, blockade of 5-HT_2A_R may offer a feasible way to suppress drug craving and/or relapse. It will be very interesting to identify the corresponding signaling pathways by which 5-HT_2A_Rs modulate these behavioral capacities. Of particular note, we reviewed evidence that 5-HT_2A_Rs may dimerize with other receptors, and that certain pathways may promote constitutive activation of 5-HT_2A_Rs, which likely represent novel receptor signaling influences. Connecting such novel properties of 5-HT_2A_Rs to distinct functional consequences of 5-HT-, or agonist-, specific activation of the 5-HT_2A_Rs will be important for improving understanding the myriad influences of 5-HT_2A_Rs in the CNS. The development of highly selective 5-HT_2A_R ligands will be essential for further establishing the critical involvement of the 5-HT_2A_R for a number of fundamental cognitive behaviors.

## Conflict of Interest Statement

The authors declare that the research was conducted in the absence of any commercial or financial relationships that could be construed as a potential conflict of interest.

## Footnotes

1 https://clinicaltrials.gov/ct2/show/NCT00207064?term=5-HT2A&rank=2

2 https://clinicaltrials.gov/ct2/show/NCT00070694?term=5-HT2A&rank=5

3 https://clinicaltrials.gov/ct2/show/NCT00086294?term=5-HT2A&rank=12

4 https://clinicaltrials.gov/ct2/show/NCT02035553?term=5-HT2A&rank=41

5 https://clinicaltrials.gov/ct2/show/NCT00477672?term=5-HT2A&rank=46

## Abbreviations

5-HT: 5-hydroxytryptamine/serotonin
AD: Alzheimer disease
AMPAR: alpha-amino-3-hydroxy-5-methyl-4-isoxazole propionate receptor
BLA: basolateral amygdala
CS: conditioned stimulus
DOI: 1-(2,5-dimethoxy-4-iodophenyl)-2-aminopropane
ERK: extracellular signal-regulated kinases
GFAP: glial fibrillary acidic protein
GPCR: G protein-coupled receptor
IP2: inositol phosphate
LSD2: lysergic acid diethylamide
mGluR2: metabotropic glutamate receptor
MUPP1: multiple PDZ protein-1
MWM: Morris water maze
NAc: nucleus accumbens
NMDAR: *N*-methyl-D-aspartate receptor
NOR: novel object recognition
OCD: obsessive–compulsive disorder
PDZ: postsynaptic density zone
PFC: prefrontal cortex
PKC: protein kinase C
PLC: phospholipase C
PSD: postsynaptic density
RSK2: ribosomal S6 kinase 2
sIPSC: spontaneous inhibitory postsynaptic current
sIPSP: spontaneous inhibitory postsynaptic potential
US: unconditioned stimulus
